# Crystal structure of *N*-[(*E*)-(1,3-benzodioxol-5-yl)­methyl­idene]-4-chloro­aniline

**DOI:** 10.1107/S1600536814022892

**Published:** 2014-10-24

**Authors:** J. Pablo García-Merinos, Yliana López, J. Betzabe González-Campos, Judit A. Aviña-Verduzco, Rosa E. del Río, Rosa Santillan

**Affiliations:** a Instituto de Investigaciones Químico-Biológicas, Universidad Michoacana de San Nicolás de Hidalgo, Morelia, Michoacán, CP 58000, México; b Departamento de Química, CINVESTAV-IPN, Apdo. Postal 14-740, 07000 México, D.F., México

**Keywords:** crystal structure, O⋯Cl contact, Schiff base

## Abstract

In the title compound, C_14_H_10_ClNO_2_, obtained by the condensation of 4-chloro­aniline and piperonal, the five-membered ring is almost planar (r.m.s. deviation = 0.023 Å) and the dihedral angle between the aromatic rings is 43.22 (14)°. In the crystal, a short O⋯Cl contact of 3.173 (2) Å is observed. The mol­ecules are arranged into corrugated (010) layers.

## Related literature   

Schiff bases have applications in fields, such as organic synthesis (Meyer *et al.*, 2007 ▶), catalysis (Itsuno *et al.*, 1990 ▶), materials science (Sliwa *et al.*, 2008 ▶), supra­molecular (Sreenivasulu *et al.*, 2012 ▶) and coordination chemistry (Drozdzak *et al.*, 2005 ▶; MacLachlan *et al.*, 1996 ▶). They display a broad spectrum of biological (Garavelli *et al.*, 1997 ▶; Ren *et al.*, 2002 ▶) and pharmacological properties, such as anti­bacterial, analgesic, anti­pyretic, anti-inflammatory and anti­cancer activities and can act as plant-growth regulators (Prakash *et al.*, 2011 ▶ and Gaur 2003 ▶). For related structures, see: Tahir *et al.* (2010*a* ▶,*b* ▶). For further synthetic details, see: Rodríguez *et al.* (2007 ▶); Domínguez *et al.* (2011 ▶).
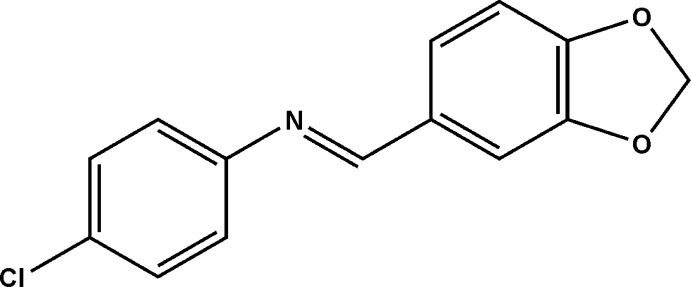


## Experimental   

### Crystal data  

C_14_H_10_ClNO_2_
*M* *_r_* = 259.69
Orthorhombic, 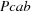

*a* = 6.0014 (4) Å
*b* = 13.9015 (16) Å
*c* = 28.867 (3) Å
*V* = 2408.3 (4) Å^3^
*Z* = 8
Mo *K*α radiation
μ = 0.31 mm^−1^
*T* = 293 K
0.19 × 0.10 × 0.08 mm

### Data collection  

Nonius KappaCCD diffractometer
5318 measured reflections
2377 independent reflections
882 reflections with *I* > 2σ(*I*)
*R*_int_ = 0.079

### Refinement  

*R*[*F* ^2^ > 2σ(*F* ^2^)] = 0.058
*wR*(*F* ^2^) = 0.121
*S* = 0.88
2377 reflections
203 parameters
All H-atom parameters refined
Δρ_max_ = 0.14 e Å^−3^
Δρ_min_ = −0.14 e Å^−3^

### 

Data collection: *COLLECT* (Nonius, 1999 ▶); cell refinement: *HKL* *SCALEPACK* (Otwinowski & Minor 1997 ▶); data reduction: *HKL* *DENZO* (Otwinowski & Minor 1997 ▶) and *SCALEPACK*; program(s) used to solve structure: *SHELXS97* (Sheldrick 2008 ▶); program(s) used to refine structure: *SHELXL97* (Sheldrick, 2008 ▶); molecular graphics: *ORTEP-3 for Windows* (Farrugia, 2012 ▶); software used to prepare material for publication: *WinGX* publication routines (Farrugia, 2012 ▶).

## Supplementary Material

Crystal structure: contains datablock(s) I, global. DOI: 10.1107/S1600536814022892/hb7302sup1.cif

Structure factors: contains datablock(s) I. DOI: 10.1107/S1600536814022892/hb7302Isup2.hkl

Supporting information file. DOI: 10.1107/S1600536814022892/hb7302Isup3.cml

**Fig. 1.**
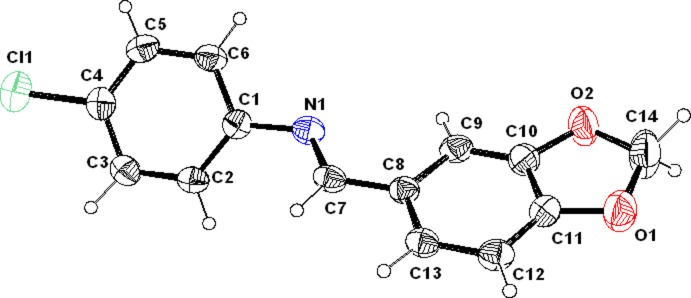
View of (I), with displacement ellipsoids drawn at 30% probability level.

I . DOI: 10.1107/S1600536814022892/hb7302fig1.tif
View of (**I**), with displacement ellipsoids drawn at 30% probability level.

CCDC reference: 1029773

Additional supporting information:  crystallographic information; 3D view; checkCIF report

## supplementary crystallographic information

### S1. Comment

Schiff bases are some of the most widely used organic compounds. They are important due to successful applications in several fields, such as organic synthesis (Meyer *et al.*, 2007), catalysis (Itsuno *et al.*, 1990) and materials science (Sliwa, *et al.*, 2008), supramolecular chemistry (Sreenivasulu *et al.*, 2012), coordination chemistry (Drozdzak *et al.*, 2005 and MacLachlan *et al.*, 1996), as well as for the broad spectrum of biological (Garavelli *et al.*, 1997 and Ren *et al.*, 2002) and pharmacological properties, such as antibacterial, analgesic, antipyretic, anti-inflammatory, anticancer, and as plant growth regulators (Prakash *et al.*, 2011 and Gaur 2003).

In previous studies, we have described an X-ray diffraction and spectroscopic study of the ketoenol tautomeric forms of six enaminones prepared from salicylaldehyde and substituted anilines (Rodríguez *et al.*, 2007). In addition we have reported a spectroscopic study of several ortho-hydroxy Schiff bases; the corresponding crystal structures were analyzed to identify their characteristic hydrogen bonding patterns, which was necessary in order to have evidence about the influence (electronic and/or structural) of the substituents on the tautomeric structure from a crystallographic perspective (Domínguez *et al.*, 2011). To continue our studies on Schiff base ligands, we synthesized the title compound (I) obtained by condensation of 4-chloroaniline and piperonal.

The dihedral angle between the two aromatic rings is 43.22 (14)° and the C1—N1—C7—C8 torsion angle is -179.0 (3)° (Table 1). The C4—Cl1 and C7=N1 bond distances are 1.716 (4) Å and 1.260 (4) Å, respectively (Table 1). These values are slightly shorter than the average values reported for Car—Cl C8=N1, 1.283 (5) Å (Tahir *et al.*, 2010*a*) and for C8=N1, 1.271 (2) Å in related Schiff bases containing the piperonal fragment (Tahir *et al.*, 2010*b*).

### S2. Experimental

A solution of 4-chloroaniline (0.500 g, 3.9 mmol) and piperonal (0.260 g, 3.01 mmol) in methanol (55 mL) was heated under reflux for 3h, with a Dean-Stark apparatus used for the azeotropic removal of water and allowed to cool to room temperature. Removal of solvent affords compound I as a pale yellow solid which was washed with hexane to obtain the product in 36% yield (m.p. 347-349 K). Colourless blocks were grown by slow evaporation from a solvent mixture of methanol:ethyl acetate (1:1). Spectroscopic data for the title compound are given in the archived CIF.

### S3. Refinement

All H atoms were found in difference Fourier maps and refined freely.

### Crystal data

**Table:**

C_14_H_10_ClNO_2_	*D*_x_ = 1.432 Mg m^−^^3^
*M**_r_* = 259.69	Melting point: 347(2) K
Orthorhombic, *P**c**a**b*	Mo *K*α radiation, λ = 0.71073 Å
Hall symbol: -P 2bc 2ac	Cell parameters from 600 reflections
*a* = 6.0014 (4) Å	θ = 2.9–27.7°
*b* = 13.9015 (16) Å	µ = 0.31 mm^−^^1^
*c* = 28.867 (3) Å	*T* = 293 K
*V* = 2408.3 (4) Å^3^	Block, colourless
*Z* = 8	0.19 × 0.10 × 0.08 mm
*F*(000) = 1072	

### Data collection

**Table:**

Nonius KappaCCD diffractometer	882 reflections with *I* > 2σ(*I*)
Radiation source: fine-focus sealed tube	*R*_int_ = 0.079
Graphite monochromator	θ_max_ = 27.7°, θ_min_ = 2.9°
φ and ω scans	*h* = −7→5
5318 measured reflections	*k* = −18→8
2377 independent reflections	*l* = −33→37

### Refinement

**Table:**

Refinement on *F*^2^	Primary atom site location: structure-invariant direct methods
Least-squares matrix: full	Secondary atom site location: difference Fourier map
*R*[*F*^2^ > 2σ(*F*^2^)] = 0.058	Hydrogen site location: inferred from neighbouring sites
*wR*(*F*^2^) = 0.121	All H-atom parameters refined
*S* = 0.88	*w* = 1/[σ^2^(*F*_o_^2^) + (0.0356*P*)^2^] where *P* = (*F*_o_^2^ + 2*F*_c_^2^)/3
2377 reflections	(Δ/σ)_max_ < 0.001
203 parameters	Δρ_max_ = 0.14 e Å^−^^3^
0 restraints	Δρ_min_ = −0.14 e Å^−^^3^

### Special details

**Table:**

Experimental. Spectroscopic data for the title compound: IR(ATR) ν_max_ cm^-1^: 2894 (CH_2_), 1600 (C=N), 1270 (C-O-C), 1490 (C=C), 827 (aromatic C-H), 789 (C-Cl) ; MS, (DIP 70 eV) for C_14_H_10_ClNO_2_*m*/*z*: (%): 259([*M*^+^],100), 261 ([*M*^+2^], 32), 138 (19), 121 (21), 75 (86). _1_H NMR (400 MHz, CDC_l3_) δ: 8.30 (s, 1H, H-7), 7.51 (d, *J*= 1.6 Hz, 1H, H-9), 7.34 (d, *J*= 8.8 Hz, 2H,H-5,3), 7.26 (dd, *J*= 8.0, 1.6 Hz, 1H, H-13), 7.12 (d, *J*= 8.8 Hz, 2H, H-6,2), 6.88 (d, *J*= 8.0 Hz, 1H, H-12), 6.04 (s, 2H, H-14).^13^C NMR (100 MHz, CDCl_3_)δ: 159.70 (C-7), 150.69 (C-10), 150.44 (C-11), 148.45 (C-8), 131.12 (C-4), 130.87 (C-1), 129.16 (C-5,3), 125.93 (C-13), 122.17 (C-6,2), 108.22 (C-12), 106.77 (C-9), 101.65 (C-14).
Geometry. All s.u.'s (except the s.u. in the dihedral angle between two l.s. planes) are estimated using the full covariance matrix. The cell s.u.'s are taken into account individually in the estimation of s.u.'s in distances, angles and torsion angles; correlations between s.u.'s in cell parameters are only used when they are defined by crystal symmetry. An approximate (isotropic) treatment of cell s.u.'s is used for estimating s.u.'s involving l.s. planes.
Refinement. Refinement of F^2^ against ALL reflections. The weighted R-factor wR and goodness of fit S are based on F^2^, conventional R-factors R are based on F, with F set to zero for negative F^2^. The threshold expression of F^2^ > 2σ(F^2^) is used only for calculating R-factors(gt) etc. and is not relevant to the choice of reflections for refinement. R-factors based on F^2^ are statistically about twice as large as those based on F, and R- factors based on ALL data will be even larger.

### Fractional atomic coordinates and isotropic or equivalent isotropic displacement parameters (Å^2^)

**Table:**

	*x*	*y*	*z*	*U*_iso_*/*U*_eq_	
Cl1	1.12362 (17)	0.14475 (7)	0.07300 (4)	0.0934 (5)	
O1	0.5831 (4)	0.12741 (17)	0.47355 (10)	0.0907 (12)	
O2	0.9308 (4)	0.07606 (17)	0.45147 (10)	0.0896 (11)	
N1	0.9505 (4)	0.10652 (15)	0.27165 (11)	0.0565 (10)	
C1	0.9840 (5)	0.11197 (19)	0.22375 (14)	0.0501 (14)	
C2	0.8314 (6)	0.0822 (2)	0.19112 (17)	0.0583 (14)	
C3	0.8719 (6)	0.0919 (2)	0.14533 (17)	0.0630 (14)	
C4	1.0729 (6)	0.13107 (19)	0.13112 (13)	0.0593 (14)	
C5	1.2271 (6)	0.1580 (2)	0.16245 (17)	0.0643 (16)	
C6	1.1841 (5)	0.1488 (2)	0.20827 (18)	0.0593 (14)	
C7	0.7604 (6)	0.1239 (2)	0.28814 (15)	0.0550 (14)	
C8	0.7055 (5)	0.12225 (18)	0.33637 (13)	0.0477 (14)	
C9	0.8629 (6)	0.0938 (2)	0.36895 (15)	0.0570 (14)	
C10	0.8071 (6)	0.0976 (2)	0.41295 (16)	0.0623 (14)	
C11	0.5986 (6)	0.1287 (2)	0.42683 (15)	0.0617 (14)	
C12	0.4405 (6)	0.1554 (2)	0.39631 (15)	0.0613 (14)	
C13	0.4983 (5)	0.1521 (2)	0.35034 (15)	0.0560 (14)	
C14	0.7954 (9)	0.0974 (6)	0.4903 (2)	0.110 (3)	
H2	0.697 (5)	0.0546 (18)	0.1998 (10)	0.066 (10)*	
H3	0.772 (5)	0.073 (2)	0.1208 (12)	0.082 (11)*	
H5	1.351 (4)	0.1855 (16)	0.1520 (10)	0.046 (8)*	
H6	1.279 (5)	0.1725 (16)	0.2307 (11)	0.059 (10)*	
H7	0.649 (4)	0.1402 (15)	0.2697 (10)	0.037 (8)*	
H9	0.994 (4)	0.0776 (16)	0.3586 (10)	0.042 (8)*	
H12	0.296 (5)	0.180 (2)	0.4049 (11)	0.079 (10)*	
H13	0.389 (5)	0.1706 (17)	0.3261 (11)	0.065 (9)*	
H14	0.851 (10)	0.150 (3)	0.507 (2)	0.20 (3)*	
H14A	0.770 (6)	0.042 (2)	0.5050 (16)	0.115 (19)*	

### Atomic displacement parameters (Å^2^)

**Table:**

	*U*^11^	*U*^22^	*U*^33^	*U*^12^	*U*^13^	*U*^23^
Cl1	0.1101 (8)	0.1037 (8)	0.0663 (8)	−0.0003 (6)	0.0110 (7)	0.0115 (6)
O1	0.075 (2)	0.132 (2)	0.065 (2)	0.0176 (14)	0.0076 (17)	0.0032 (16)
O2	0.0707 (17)	0.147 (2)	0.051 (2)	0.0218 (14)	−0.0070 (17)	0.0085 (16)
N1	0.0407 (17)	0.0619 (16)	0.067 (2)	0.0055 (11)	0.0016 (15)	−0.0008 (14)
C1	0.046 (2)	0.0462 (19)	0.058 (3)	0.0047 (14)	0.004 (2)	−0.0004 (16)
C2	0.051 (2)	0.056 (2)	0.068 (3)	−0.0105 (16)	−0.004 (2)	−0.003 (2)
C3	0.063 (2)	0.066 (2)	0.060 (3)	−0.0056 (18)	−0.010 (2)	−0.007 (2)
C4	0.068 (2)	0.053 (2)	0.057 (3)	0.0078 (17)	0.001 (2)	0.0004 (17)
C5	0.050 (2)	0.065 (2)	0.078 (4)	−0.0065 (17)	0.007 (2)	0.006 (2)
C6	0.043 (2)	0.068 (2)	0.067 (3)	0.0002 (17)	−0.007 (2)	−0.006 (2)
C7	0.046 (2)	0.052 (2)	0.067 (3)	0.0027 (15)	−0.016 (2)	0.0053 (18)
C8	0.043 (2)	0.0461 (19)	0.054 (3)	0.0013 (14)	−0.0044 (18)	0.0058 (16)
C9	0.040 (2)	0.064 (2)	0.067 (3)	0.0043 (16)	0.009 (2)	0.0027 (19)
C10	0.055 (2)	0.069 (2)	0.063 (3)	0.0027 (16)	−0.001 (2)	0.005 (2)
C11	0.059 (2)	0.071 (2)	0.055 (3)	−0.0004 (17)	0.007 (2)	0.001 (2)
C12	0.046 (2)	0.066 (2)	0.072 (3)	0.0088 (18)	0.003 (2)	−0.004 (2)
C13	0.044 (2)	0.060 (2)	0.064 (3)	0.0032 (15)	−0.005 (2)	0.0007 (19)
C14	0.098 (4)	0.169 (7)	0.062 (4)	0.034 (4)	0.003 (3)	0.012 (4)

### Geometric parameters (Å, º)

**Table:**

Cl1—C4	1.716 (4)	C8—C13	1.372 (4)
O1—C11	1.352 (5)	C9—C10	1.315 (6)
O1—C14	1.425 (6)	C10—C11	1.383 (5)
O2—C10	1.370 (5)	C11—C12	1.347 (5)
O2—C14	1.416 (6)	C12—C13	1.372 (6)
N1—C1	1.399 (5)	C2—H2	0.93 (3)
N1—C7	1.260 (4)	C3—H3	0.96 (3)
C1—C2	1.377 (5)	C5—H5	0.89 (2)
C1—C6	1.380 (4)	C6—H6	0.92 (3)
C2—C3	1.351 (7)	C7—H7	0.88 (3)
C3—C4	1.386 (5)	C9—H9	0.87 (2)
C4—C5	1.347 (6)	C12—H12	0.97 (3)
C5—C6	1.354 (7)	C13—H13	0.99 (3)
C7—C8	1.431 (6)	C14—H14	0.94 (5)
C8—C9	1.390 (5)	C14—H14A	0.89 (3)
			
C11—O1—C14	106.3 (3)	C11—C12—C13	116.4 (3)
C10—O2—C14	106.6 (3)	C8—C13—C12	121.6 (3)
C1—N1—C7	119.6 (3)	O1—C14—O2	107.8 (4)
N1—C1—C2	124.3 (3)	C1—C2—H2	121.2 (18)
N1—C1—C6	117.7 (3)	C3—C2—H2	117.5 (18)
C2—C1—C6	118.0 (4)	C2—C3—H3	125 (2)
C1—C2—C3	121.3 (3)	C4—C3—H3	116 (2)
C2—C3—C4	119.1 (4)	C4—C5—H5	117.8 (19)
Cl1—C4—C3	119.2 (3)	C6—C5—H5	122.1 (19)
Cl1—C4—C5	120.3 (3)	C1—C6—H6	116.3 (19)
C3—C4—C5	120.6 (4)	C5—C6—H6	122.3 (19)
C4—C5—C6	119.9 (3)	N1—C7—H7	120.4 (18)
C1—C6—C5	121.2 (4)	C8—C7—H7	114.6 (18)
N1—C7—C8	125.0 (3)	C8—C9—H9	117.1 (19)
C7—C8—C9	120.4 (3)	C10—C9—H9	124.9 (19)
C7—C8—C13	119.3 (3)	C11—C12—H12	124.3 (19)
C9—C8—C13	120.2 (4)	C13—C12—H12	119.2 (19)
C8—C9—C10	118.0 (3)	C8—C13—H13	118.0 (18)
O2—C10—C9	129.6 (3)	C12—C13—H13	120.4 (18)
O2—C10—C11	108.9 (4)	O1—C14—H14	105 (3)
C9—C10—C11	121.5 (4)	O1—C14—H14A	105 (2)
O1—C11—C10	110.3 (3)	O2—C14—H14	112 (4)
O1—C11—C12	127.4 (3)	O2—C14—H14A	107 (3)
C10—C11—C12	122.3 (4)	H14—C14—H14A	119 (4)
			
C14—O1—C11—C12	−177.8 (4)	C3—C4—C5—C6	−1.5 (4)
C11—O1—C14—O2	−3.3 (6)	C4—C5—C6—C1	0.1 (4)
C14—O1—C11—C10	2.2 (5)	N1—C7—C8—C9	−4.5 (4)
C14—O2—C10—C11	−1.7 (4)	N1—C7—C8—C13	173.3 (3)
C14—O2—C10—C9	176.9 (4)	C13—C8—C9—C10	−0.7 (4)
C10—O2—C14—O1	3.1 (6)	C9—C8—C13—C12	0.4 (4)
C7—N1—C1—C6	142.4 (3)	C7—C8—C9—C10	177.1 (3)
C7—N1—C1—C2	−38.2 (4)	C7—C8—C13—C12	−177.5 (3)
C1—N1—C7—C8	−179.0 (3)	C8—C9—C10—O2	−178.4 (3)
C2—C1—C6—C5	1.8 (4)	C8—C9—C10—C11	0.1 (4)
N1—C1—C6—C5	−178.8 (3)	O2—C10—C11—O1	−0.4 (3)
N1—C1—C2—C3	178.2 (3)	O2—C10—C11—C12	179.7 (3)
C6—C1—C2—C3	−2.5 (4)	C9—C10—C11—O1	−179.1 (3)
C1—C2—C3—C4	1.2 (4)	C9—C10—C11—C12	1.0 (5)
C2—C3—C4—Cl1	−179.3 (2)	O1—C11—C12—C13	178.8 (3)
C2—C3—C4—C5	0.8 (4)	C10—C11—C12—C13	−1.3 (4)
Cl1—C4—C5—C6	178.7 (2)	C11—C12—C13—C8	0.6 (4)
