# Supplementary figures and images for: Crystal structure of N-[(E)-(1,3-benzodioxol-5-yl)­methyl­idene]-4-chloro­aniline
Source: Acta Crystallogr Sect E Struct Rep Online. 2014 Oct 24;70(Pt 11):o1195–6. doi: 10.1107/S1600536814022892 (PMC4257285; doi:10.1107/S1600536814022892)

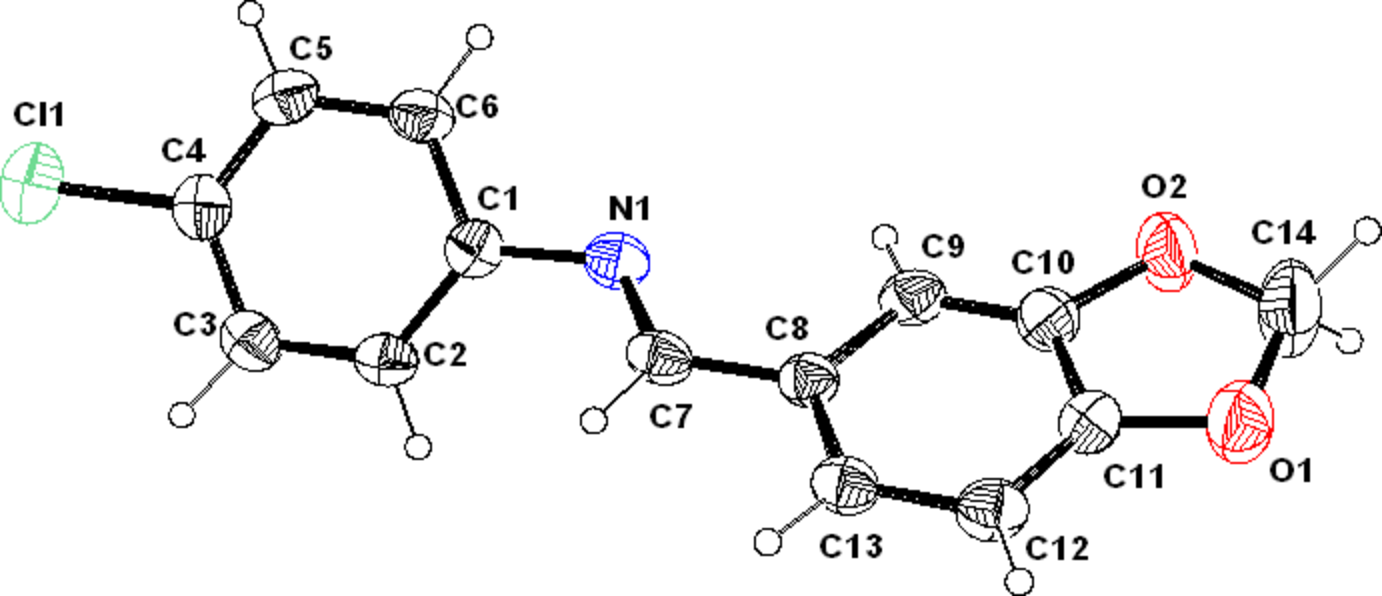

Supplement: Supplementary file 4 [file e-70-o1195-fig1.tif]
